# Core Outcome Sets and Multidimensional Assessment Tools for Harmonizing Outcome Measure in Chronic Pain and Back Pain

**DOI:** 10.3390/healthcare4030063

**Published:** 2016-08-29

**Authors:** Ulrike Kaiser, Katrin Neustadt, Christian Kopkow, Jochen Schmitt, Rainer Sabatowski

**Affiliations:** 1 Comprehensive Pain Center, University Hospital “Carl Gustav Carus”, Technical University Dresden, Dresden 01307, Germany; katrin.neustadt@uniklinikum-dresden.de (K.N.); rainer.sabatowski@uniklinikum-dresden.de (R.S.); 2 Center for Evidence-Based Healthcare, Medical Faculty, Technical University Dresden, Dresden 01307, Germany; christian.kopkow@uniklinikum-dresden.de (C.K.); jochen.schmitt@uniklinikum-dresden.de (J.S.); 3 Department of Anesthesiology and Intensive Care, University Hospital “Carl Gustav Carus“,Technical University Dresden, Dresden 01307, Germany

**Keywords:** core outcome set, effectiveness, efficacy, pain management, chronic pain, back pain, daily record keeping, clinical trials

## Abstract

Core Outcome Sets (COSs) are a set of domains and measurement instruments recommended for application in any clinical trial to ensure comparable outcome assessment (both domains and instruments). COSs are not exclusively recommended for clinical trials, but also for daily record keeping in routine care. There are several COS recommendations considering clinical trials as well as multidimensional assessment tools to support daily record keeping in low back pain. In this article, relevant initiatives will be described, and implications for research in COS development in chronic pain and back pain will be discussed.

## 1. Introduction

Chronic pain, especially non-specific chronic low back pain (NLBP), is a frequently encountered phenomenon with considerable psychosocial and overall socio-economic consequences. In recent decades, clinical and health care service research has provided substantial international contribution to several approaches in pain management. Particularly in relation to NLBP and interdisciplinary multidisciplinary pain therapy (IMPT), numerous studies formed the basis for a large number of systematic reviews and meta-analyses (e.g., [1,2,3,4]). However, there are still unsolved problems in analyzing IMPT such as the heterogeneity of outcome assessment in clinical trials and interventional studies which hamper drawing conclusions out of those studies and/or systematic reviews. e.g., for multidisciplinary pain therapy systematic reviews express the need for a standardized use of outcome parameters for measuring treatment success in those programs, and for a consideration of reliability and validity of measuring instruments. This leads to significant limitations in the interpretability of results. The problems observed in integrating results on a meta-perspective are exemplarily for most of the systematic reviews and meta-analyses at the moment [5,6,7].

## 2. Developing COS for Clinical Trials-Introduction to Method and Development

Establishing Core Outcome Sets is recommended to overcome such limitations and to enable researchers to integrate data in systematic reviews and meta-analyses. A Core Outcome Set (COS) is defined as a minimum set of outcome domains, which are recommended to be applied in each clinical trial and to be extended by other domains according to the specific study design [6]. Some authors extend the definition of COS including further relevant, reliable and valid measurement instruments as well [8]. The development of a COS has once been pioneered by OMERACT (Outcome Measures in Rheumatology; [5]). First guidance in developing COS has been presented by HOME (Harmonizing Outcome Measures in Eczema; [8]) and COMET (Core Outcome Measures in Effectiveness Trials; [6]). According to Schmitt et al. [8] developing COSs consists of several different steps, frequently beginning with a systematic review of all outcomes reported in clinical trials and a subsequent consensus process to vote for relevant outcome domains which should be assessed in clinical trials. Of high importance are relevant and important stakeholders joining the expert panel, including patient representatives who are expected to best decide about relevant outcomes [9]. Online surveys are common for achieving consensus, but still the methodology of COS development is various and heterogeneous [10]. Alongside the discussion of COS domain development, psychometric properties of measurement instruments to measure COS domains have been questioned and guidelines have been developed by COSMIN (COnsensus-based Standards for the selection of health Measurement Instruments; [11,12,13,14]). Further, outcome measures of COS should adequately meet the criteria of truth (i.e., validity; measure what they intend to measure), discrimination (i.e., reliability and sensitivity to change; discriminate between situations), and feasibility (i.e., be applied and interpreted easily) in order to be meaningful and relevant [15]. According to the notion that studies are only as credible as their outcome measures [15] measures have to be validated on target population [13]. This is always an important issue especially when considering comprehensive therapy approaches and/or heterogeneous patient populations.

Naturally standardization in developing COS consisting of relevant domains and valid and reliable measurement instruments is work in progress. Updates will become necessary due to advances in research, therapy provision, quality of conceptual definitions and measurement instruments.

## 3. Core Outcome Sets for Low Back Pain in Clinical Trials

Based on the described obstacles in practicing evidence-based medicine, some outcome initiatives with special focus on chronic pain in general [16,17] and non-specific low back pain [18,19,20,21,22,23] have been established. The main objective of these initiatives is to recommend a consensus on COS of outcome domains and measures that should be used in each clinical trial to enable comparison estimates of the benefits of different pain interventions (e.g., medication, surgery). An overview of the different recommendations is provided in Table 1.

The IMMPACT initiative (Initiative on Methods, Measurement, and Pain Assessment in Clinical Trials) recommended 6 outcome domains to be included in any clinical trial of therapy approaches in chronic pain in general, including NLBP: pain, physical functioning, emotional functioning, participant’s ratings of global improvement, symptoms and adverse events, and participant’s disposition (including adherence to the treatment regimen and reasons for premature withdrawal from the trial) [16]. Additional domains were recommended to be assessed optionally according to study question and aim (role functioning, interpersonal functioning, pharmacoeconomic measures and health care utilization, biological markers, coping, clinician or surrogate ratings of global improvement, neuropsychological assessments of cognitive and motor function, and suffering and other end of life issues). Panel members of IMMPACT consisted of different professions (see Table 1). However, patient representatives had not been included [16]. A survey performed with patients suffering from chronic pain indicated other outcome domains as compared to the first recommendations [16,24]. Patients rated the domains sleep, sexual activities, ability to fulfill role function, work ability, several forms of activities (physical, homework, work, and social activities), emotional wellbeing, weakness and fatigue, and cognitive impairment to be obligatory in assessing therapy effectiveness [24]. The patient relevant outcome domains are in accordance with the additional recommendations of IMMPACT [16], but not with the main recommendation (see Table 1).

Alongside these recommendations for chronic pain in general, there are others which are more disease specific. Especially for non-specific low back pain a long history of attempts to standardize outcome exists [18,19,20,21,22,23]. Quite recently, an update of a former recommendation by Deyo [23] for NLBP (consisting of pain symptoms, function, well-being, disability (physical and social roles) and satisfaction with care) was published by Chiarotto et al. [18]. A group of 280 researchers of different professions and backgrounds, patients and health care providers guided by a steering committee was led through a complex Delphi process with clearly specified definition of consensus. Starting with 41 outcome domains derived from systematic reviews in 3 Delphi rounds (response rates 45%–52%) finally 3 domains were recommended to be COS relevant: physical functioning, pain intensity and health related quality of life, whereby health related quality of life was not supported by the patient group. The steering committee decided to include an additional domain “number of deaths” (as recommended by OMERACT [15]) into the COS even though they stated occurring death in clinical trials in NLBP to be a rare event. The COS is assumed to serve for all clinical trials in NLBP. All domains were accompanied by a consented definition. Defining measurement instruments is now work in progress and will complete the recommendation [18]. Other initiatives for NLBP recommended further overlapping or distinct outcome domains by different kinds of decision making processes [21,22]. They mainly included clinicians and researchers to identify relevant outcome domains.

A setting specific approach for vocational rehabilitation of NLBP and musculoskeletal pain patients in the Netherlands is pursued by Reneman et al. [20] who developed a COS integrating ICF (International Classification of Function) for low back pain [19] and IMMPACT [16] recommendations, resulting in 18 outcome domains assessed by 12 measurement instruments. Reneman et al. kept the ICF framework and extended it by primary and supplemental outcome domains as recommended by IMMPACT. Patient participation in the process of defining COS was not considered and the panel consisted mainly of physicians specialized in rehabilitation medicine. Psychometric properties of measurement instruments were discussed as satisfactory [20]. Recommended domains are provided in Table 1.

Since the therapy of chronic pain can pursue different aims the question emerged to what extent a more unspecific recommendation, e.g., IMMPACT recommendation, can be applied to a specific therapy approach in chronic pain. The VAPAIN initiative (Validation and Application of patient reported outcome domains to assess in multimodal pain therapy) targets to assessing effectiveness of an interdisciplinary multimodal therapy (IMPT) of chronic pain [17]. The project is a comprehensive and multi-method approach consisting of several steps of systematic reviews (domains [25], instruments (in preparation)), a multistep consensus process on domains and instruments accomplished by validation studies investigating psychometric properties of potential instruments. According to previous recommendations [9] panelists experienced in IMPT or COS development and with international and multi-professional background (consisting of patient representatives, physicians specialized in pain medicine, physiotherapists, psychotherapists and methodological experts) were invited. The challenge of VAPAIN is the biopsychosocial model of chronic pain as a fundamental basis of the chosen therapy approach, leading to a complex intervention. This means that all future included outcome domains shall cover biological, psychological and social aspects affected by chronic pain.

## 4. COS Measurement Instruments to Be Applied in Chronic Pain

Application of COS requires associated measurement instruments. For the purpose of assessment in pain therapy there is a broad variety of measurement instruments, covering many aspects of a biopsychosocial model of chronic pain. Deckert et al. identified more than 140 outcome domains in the setting of IMPT [33], but even more applied instruments limiting comparisons between studies and meta-analyses. e.g., pain intensity was measured in 56 out of 70 included studies, the variety of the different instruments and their presentation was considerable (e.g., time period, interval of Likert-scales, specific categories of pain levels etc.) [33]. Currently the psychometric properties of measurement instruments for pain intensity are critically reflected [34,35,36].

IMMPACT proposed measurement instruments for their primary outcome recommendation [26]. The authors reported that psychometric property particularly of the psychological scales (e.g., Beck Depression Inventory, Profile of Mood States) was lacking or insufficient. Despite of this problem and due to the absence of alternatives between one and three measurement instruments for each domain were recommended (Table 1).

In a recently published overview representatives of IMMPACT and OMERACT discussed existing measurement instruments for physical function and participation [37]. The authors reported a considerable variety of such instruments but still open questions for example according to the discrepancies between patient reported outcome (PRO) instruments and objective measures of physical function and influencing psychosocial factors. The need for PROs and inclusion of patient representatives into developmental processes for PROs assessing physical function and participation was repeatedly emphasized [37].

The functional barometer [38] has been developed as a measurement tool to assess ICF criteria in patients with long term pain accompanied by pain related problems with function, activity and quality of life. It consists of items for patient reporting and correspondingly a classification form for professionals to assess patients’ problems from the clinicians’ perspective. Norrefalk reported a significantly underestimation of the patients’ perceived problems followed by a large variability between the different observers, and assumed that integrating the patients’ perception of pain related problems should be regarded as to be of high value within the assessment in clinical trials [38]. A review by Jelsma [39] demonstrated that ICF was broadly applied, but main critic refers to complication in coding pain and the lack of codes for personal factors (such as satisfaction with specific aspects, personal experience or emotional states).

Ashburn et al. [40] highlights that lacking data may put the specialty of pain medicine at risk and calls researchers to redouble the efforts “to demonstrate that what we do, in fact, matters- and that the care we provide improves the lives of those we serve as well as society as a whole” [40]. One way to do so is to clear up the situation of heterogeneous and therefore incomparable outcome domains and measurement tools to enhance meta-analyses. This also includes a careful work on psychometric properties of measurement instruments in pain therapy, consequently considering the characteristics and specialties of its very heterogeneous population. It is necessary to acknowledge the requirements of the process of investigating instruments as well as the amount of resources and effort to ensure high validity and reliability of concepts and instruments in pain therapy.

## 5. Core Outcome Sets for Daily Record Keeping in Routine Care for Patients with Back Pain

Several initiatives have worked on recommendation and standardization on outcome assessment in daily record keeping (DRK; [41,42,43,44,45,46], see Table 2). The German Pain Questionnaire [41,42] is provided to all specialized pain centers throughout Germany and supports quality management of the diagnostic and therapeutic process. Via an electronic platform benchmarking for each institution is possible. To fulfill requirements of diagnosis and therapy in different settings (outpatients, inpatients, specific approaches in pain therapy) the included variables are comprehensive comprising sociodemographic data, pain variables (e.g., pain sites, temporal characteristics, duration, intensity), pain associated symptoms, affective and sensory qualities of pain, pain relieving and intensifying factors, previous treatment procedures, pain related impairment, and psychosocial factors (see Table 2). For users the authors provide normative data and cut-off points for several scales.

For multidisciplinary outpatient treatment the Treatment Outcome of Pain Survey (TOPS) has been developed and completed by norms for initial values and treatment related improvements [43,44]. A short form has been published recently [45]. Basing on the SF-36 the original TOPS-version was generated by incorporating specific additional variables following a scientific model of disablement [47], consisting of pain symptoms, functional limitations, perceived family/social disability, objective family/social disability, and objective work disability (see Table 2). To complete the biopsychosocial perspective other items concerning life control, passive coping, solicitous responses, fear avoidance, upper body functional limitations, satisfaction with care and outcomes, and work limitation have been included as well. The authors reported sufficient psychometric properties (reliability and validity). As the authors concluded, the TOPS distinguishes from other pain and quality-of–life instruments, e.g., it bases on a treatment model, it comprises both treatment and context factors and it tracks individual change as well as documents the outcomes of groups of patients [44]. Rogers furthermore recommended the time line of providing the TOPS to patients and for a fast and efficient administration process in routine clinical care [44].

Since the original TOPS consisted of 14 subscales and 8 subscales of the SF-36 a previous initiative has tried to come up with a reduced version to improve feasibility [45]. A multi-methodic approach has been conducted including judgment of experienced clinicians as well as criteria of psychometric property and patients were asked about the acceptable amount of items. Finally, seven subscales, including 4 out of 6 IMMPACT domains, were recommended (physical function lower body, physical function upper body, pain symptom, role-emotional disability, family and social disability, patient satisfaction with outcomes, patient satisfaction with care) accomplished by the SF-12 subscales replacing the former SF-36 subscales [45]. To complete the recommended set of scales Haroutiunian et al. suggested two more scales—performance/work disability scale and sleep scale [45]. The authors recommended these instruments for patient reported outcome assessment for monitoring chronic pain treatment by individual change and reported sufficient psychometric properties (reliability, validity, and sensitivity to change), emphasizing that the inclusion of IMMPACT recommendations should enhance the process of translation from research into immediate clinical practice.

A patient centered approach was presented by Casarett et al. [46], where patients were asked by qualitative interviewing and quantitative assessment about the most relevant outcome domains for medication treatment. Patients indicated 20 outcome domains, e.g., decrease pain, decrease opioid dose, decrease frequency of scheduled dose, increased ability to function, decrease frequency of breakthrough dose and improve sleep. The authors concluded, that the opinion of patients’ needs to be valued when designing studies and defining relevant outcome. The Patient Centered Outcome Questionnaire (PCOQ, [48]) targets 4 outcome domains such as pain, fatigue, emotional distress, and interference with daily activities. The origin of the chosen outcome domains unfortunately remains unclear. Notable is the focus of judging the outcome domains by the patients in 3 levels: usual level, desired level, and level of success [48]. This way therapy success is clearly defined by patients’ expectations and differs from clinicians’ definition of treatment success in chronic back pain [46].

For Germany an initiative provides another tool to picture effectiveness in daily routine care of IMPT institutions [49]. The authors selected items and scales from the German Pain Questionnaire [41,42] such as average pain intensity (NRS, 0–10), Pain Disability Index (PDI), German version of the Center of Epidemiologic Studies Depression Scale (CES-D) and the SF-36. The authors suggested a combined criterion consisting of the presented instruments and the criteria that 4 out of 5 scales should have changed at least 0.5 standard deviations to indicate a successful change. The tool was reported to be useful to identify more than 50% of patients to have recovered in at least 4 of the 5 recommended criteria [49]. The preference of the patients about the different success criteria and their cut-off had not been considered. Including the perspective of patients might have led to completely different criteria and their combination.

Regarding these different approaches it becomes obvious that each approach has focused on a specific aspect or function. Some want to support diagnostic and therapeutic process; others want to ensure high quality of array of treatment. Several issues have been picked up, such as success criteria or the distinction between individual or group change. All of these initiatives have brought up important, yet until today unsolved parts of therapy quality assessment. An overarching work would help to set the frame of definition and requirements of COS in DRK.

## 6. Issues for Further Consideration in the Discussion of COS for Chronic Pain

### 6.1. General Issues

Considering core outcome domains there is an overlap in recommended outcome domains or areas of the different initiatives on chronic pain comprising pain (intensity), physical function, and psychological factors (distress, emotional wellbeing, emotional functioning). Nevertheless there are still significant gaps between these different recommendations. Primarily, the scope of the domains varies significantly, for instance focusing on emotional functioning [16] or emotional wellbeing [24]. Even though the area of the domains is the same (psychological) the underlying concepts might be wide apart. A definition of theoretical constructs of domains was not always provided. Many of the presented initiatives have included biological and emotional areas and domains but still lack social components [16,18,22] (see Table 1). Some initiatives have tried to connect with other initiatives [20,34]. This has led to a greater overlap between the different recommendations and seems to be a promising way to close the existing gaps. For daily record keeping the recommendations are even more heterogeneous, both in recommended domains and number of domains (see Table 2). The recommendations vary according to national or international focus as well as to the setting they consider (e.g., individual patient monitoring [36] or support of therapy and diagnostic approach [38]). Different outcome measurement instruments might be a consequence and still hamper standardized outcome measurement in effectiveness studies in chronic pain as described on the example of the domain of pain intensity. From the current point of view it needs to be stated that there is still a considerable lack of valid and reliable measurement instruments or unclear evidence of psychometric properties of existing instruments. Previous reports about measurement instruments and their properties for pain intensity vary significantly, from no evidence of psychometric property for pain intensity [30], unclear evidence because of low report quality [29] to good results in psychometric property for patient reported outcome questionnaires for people with pain in any spine region while mainly fair methodological quality [51]. Lacking methodological quality is a well-known problem in the field of measurement instruments and affects most of the instruments in pain research [26]. The work of the COSMIN group is therefore promising and gratifying [11,12,14]. The basis of methodological standards need to be reinforced by thoroughly designed validation studies, starting with content validity and taking into account patients’ perspectives while designing scales [13]. Existing scales should be careful investigated according to their psychometric properties in the sample of patients with chronic pain [13]. Other aspects of applying scales and interpreting their results affect the context of assessment. Relevance and sensitivity of outcome measurement instruments might interact with acquainted active components of therapy approaches. It seems considerable that domains might be more useful when linked to an attribute targeted by therapy. For instance, depression will only consistently and consequently change according to an intervention when it is specifically aimed for. Further the requirement of patient reported outcomes (outcomes picturing domains relevant to patients) necessitates the consideration of patient aims, which depend also on the applied intervention. Further concerning DRK measurement instruments should be sensitive to individual’s change as well as to group effects. The translation of clinical results into practice as being part of treatment research needs to consider both, requirements for DRK as well as for clinical trials/effectiveness studies, which have not been discussed until today but are necessary to further establish COS in specific settings.

### 6.2. Implementing and Updating COS

Implementation of a COS as part of the complete process has been highlighted by Schmitt et al. [8]. There are at least two important issues to be considered for this step: Feasibility and content validity of a COS will certainly influence implementation. In addition to the domains which are part of a specific COS, other outcome domains can be of relevance for specific study objectives. Therefore the reasonably limited number of required COS domains shall enable researchers to add other domains and still keep the set of questionnaires feasible to use. Another important issue is the existence of competing COS recommendations as observed in chronic pain. Naturally competing COS will not solve the existent situation of incomparable studies. An initiative to bring together the different COS recommendations with focus of clinical trials to find consensus on recommended overlap and further indicators for a specific COS application might help researchers to decide which COS is appropriate for a designed clinical trial.

The application of such COS’s should not be restricted to clinical trials only. The attempt to translate the knowledge about efficacy from clinical, standardized investigation of a therapy approach into effectiveness of daily routine care needs at least an overlap of relevant domains. Therefore, a COS is also relevant within routine care [52]. None of existing initiatives focused on therapy effects of interventions for chronic pain for both effectiveness studies and daily recordkeeping in particular. Yet, for DRK an international recommendation for one COS seems to be illusory at the moment considering the different national requirements of structural and procedural characteristics of health care delivery, health care politics and grown landscape of therapy approaches.

Developing COS is work in progress. Concepts of therapy or methodological approaches change as well as the perspective of clinicians, researchers and patients. A COS will need to be updated considering advances in all those areas in a manageable time period.

## 7. Conclusions

Core Outcome Initiatives in chronic pain target on harmonizing outcome assessment in clinical trials, but frequently focus on different aspects, such as specific conditions, therapy approaches or clinical settings. Implementing COS, as proposed to be part of an extended process of COS development [8], depends on distinct indicators when to apply a specific COS, especially when competing COS exist. Implementation also requires the application of valid and reliable measurement instruments. At the moment the psychometric property of several instruments is either unknown or insufficient. The careful identification of stake holders, patient representatives and scope of a COS will strongly influence its acceptance and its implementation. Only accomplished by reliable, valid and feasible instruments a COS serves well for meta analyses in evidence based medicine.

## Figures and Tables

**Table 1:** Recommendations for core outcome sets for clinical trial and/or effectiveness studies in chronic pain and back pain (table modified and adapted from Deckert et al. [25]).

Name of Initiative/Author	(a) Condition (b) Intervention (c) Scope of application (d) Location	Core Outcome Set -Domains-	Core Outcome Set -Measurement Instruments-	Stakeholders	Additional Comments
**ICF Core sets for low back pain** Cieza et al. 2004 [19]	(a) Low back pain (b) Not reported (c) Not reported (d) International	**4 outcome domains** body functions body structures activities and participation environmental factors Different number of second level categories for a *comprehensive set* and *a brief set*	ICF category system	Panel consisted of 18 experts (3 occupational therapists, 1 physical therapists, 14 physicians with various sub-specializations)	Formal decision-making and consensus process with systematic review, Delphi exercise and empirical data collection The both sets were recommended for validation only
**ICF/IMMPACT for vocational rehabilitation** Reneman et al. 2013 [20]	Musculoskeletal pain (subacut and chronic) Vocational rehabilitation Clinical research and clinical practice Regional (The Netherlands)	**18 outcome domains** (based on IMMPACT and ICF), e.g., Quality of life Physical functioning Pain intensity Emotional functioning Coping	12 measurement instruments such as: EuroQuol-5D Pain Disability Index (PDI); RAND 36-Item Health Survey Numerical Rating Scale (NRS) Work Reintegration Questionnaire (Distress sub-scale) Work Reintegration Questionnaire (Distress Avoidance and Persistence Sub-scale)	Preliminary core set was presented to 3 groups: Dutch Vocational rehabilitation center (*n* = 13; user, clinicians, management) Dutch pain rehabilitation development centers (*n* = 4; pain rehabilitation experts) Members of the consensus group (vocational rehabilitation) (*n* = 23; vocational rehabilitation experts)	Elaborate procedure to identify relevant outcome domains and measurement instruments: 1. Domains were identified according to ICF and IMMPACT recommendations 2. Domains were classified and judged by panel and authors (also according to the use in economic evaluation) 3. Instruments were identified for the included domains according to specific requirements of psychometric property
**IMMPACT** Turk et al. 2003 [16] Dworkin et al. 2005 [26]	Chronic pain No specific Clinical trials International	**6 outcome domains** (1) Pain (2) Physical functioning (3) Emotional functioning (4) Participant’s ratings of global improvement (5) Symptoms and adverse events, and (6) Participant’s disposition **additional domains** according to study aim: - role functioning—interpersonal functioning—pharmacoeconomic measures and health care utilization, - biological markers, - coping, - clinician or surrogate ratings of global improvement—neuropsychological assessments of cognitive and motor function, and—suffering and other end of life issues	(1) 11 point (0–10) numerical rating scale of pain intensity (NRS) Usage of rescue analgesics Categorical rating of pain intensity (none, mild, moderate, severe) in circumstances in which numerical ratings may be problematic (2) Multidimensional Pain Inventory Interference Scale or Brief Pain Inventory Interference Items (3) Beck Depression Inventory (BDI) or Profile of Mood States (PMS) (4) Patient global assessment of change (PGIC) (5) Passive capture of spontaneously reported adverse events and symptoms and use of open-ended prompts (6) Detailed information regarding participant recruitment and progress through the trial, including all information specified in the CONSORT guidelines No measurement recommendations for the additional outcome domains	Domains [16] 27 participants with backgrounds in anesthesiology, biostatistics, clinical pharmacology, epidemiology, geriatrics, internal medicine, neurology, nursing, oncology, pediatric pain, physical medicine and rehabilitation, psychology, and rheumatology, all with research, clinical, or administrative expertise relevant to evaluating chronic pain treatment outcomes additionally representatives from the pharmaceutical industry and an attorney for specific expertise Measurement instruments [26] 35 participants from academia, governmental agencies, a self-help organization, and the pharmaceutical industry	Consensus process consisting of presence meeting and preselected clinical trials to identify relevant outcome domains Other issues have been published for assessing effectiveness in chronic pain, e.g.,: - Analyzing multiple endpoints [27] - Interpreting the clinical importance of group differences [28] - Interpreting the clinical importance of treatment outcomes [29] - Developing patient reported outcome measures [30] - COS for pediatric acute pain in clinical trials [31] #
**IMMPACT** **Survey with patient representatives** Turk et al. 2008 [24]	Chronic pain No specific Clinical trials International	**19 outcome domains e.g.,:** - sleep, - sexual activities, - ability to fulfill role function, - work ability, - several forms of activities (physical, homework, work, and social activities), - emotional wellbeing, weakness and fatigue, - cognitive impairment (e.g., concentrating and remembering)	Not reported	Patient representatives Preparing focus groups *n* = 31 Web survey *n* = 959	Preparing of relevant outcome domains via focus groups Validating via web survey
**Low back pain** Deyo et al. 1998 [23]	Low back pain No specific Clinical trials and other kinds of research (also routine care) International	**6 outcome domains** (1) Pain symptoms (2) (Physical) function (3) Well being (4) Disability (5) Disability (social role) (6) Satisfaction with care	**For routine clinical use, quality improvement and as a component of formal research** All domains form a set of six questions (six items), adapted from several instruments such as Short- Form 36 Questionnaire (SF-36(SF36), Roland and Morris disability scale (RMDS), EuroQuol and others **For researchers** (1) Bothersomeness or severity and frequency of low back pain and leg pain (2) Roland and Morris Disability scale (RMDS) or Oswestry Disability Questionnaire (ODQ) (3) Short- Form 12 Health Survey (SF-12) or EuroQuol (4) not mentioned (5) Days of work absenteeism, cut down activities, bed rest (6) single question on overall satisfaction (optional)	A multinational group of investigators	Consensus process not reported
**Low back pain** Bombardier 2000 [21]	Low back pain No specific Clinical and health policy setting International	**5 outcome domains** (1) Back specific function (2) Generic health status (3) Pain (4) Work disability (5) Patient satisfaction	(1) Oswestry Disability Questionnaire (ODQ) or Roland Morris Disability Questionnaire (RMDQ) (2) Short Form 36 (SF-36) (3) Bodily pain Scale (SF-36), optional Chronic pain grade (CPG) (4) Work status; #days off work and day of cut down work, # of day return to work (5) Patient satisfaction scale (PSS) and Satisfaction with treatment (one item)	Clinicians (physicians, psychologists, researchers experienced in pain medicine, outcome research and development of questionnaires)	non-formal consensus process not further described
**Low back pain** Chiarotto et al. 2015 [18]	Non-specific low back pain No specific Clinical trials International	**4 outcome domains** Physical functioning Pain intensity Health related quality of life * Number of deaths ** (for * and ** please refer to *additional comments*)	In preparation	Steering group consisting of members from four continents, including researchers, health care providers and patient representatives Panel was identified by systematic review about number of publications as an indicator for expertise, including representatives from health care researchers, health care providers, professionals working both as researchers and providers and patients with non-specific low back pain; *n* = 280	Three stage online Delphi and consensus exercise As an update of the former recommendation by Deyo et al. [23] * health related quality of life was not supported by the patient group.**Based on OMERACT 2.0 Filter framework [15]
**VAPAIN** Kaiser et al. 2015 [17]	Chronic pain Interdisciplinary multimodal pain therapy Effectiveness studies and daily record keeping International	In preparation	In preparation	Panel consists of 25 participants, 5 of each patient representatives, physicians, psychotherapists, physiotherapists with experience in interdisciplinary multimodal pain therapy and researches with methodological expertise in COS development and development of questionnaires	The VAPAIN process targets also towards the development and/or validation of measurement instruments for effectiveness studies and daily record keeping in MPT Multi-methodic consensus process with online exercises, structured consensus process and moderated face to face meeting Based on PROMIS framework [32]
**WHO back pain initiative** Ehrlich 2003 [22]	Low back pain No specific In all studies international (to all cultures)	Not specified	(1) Appropriate history and physical examination (2) Modified Schober Test of spinal mobility (3) Measurement of pain via visual analogue scale (4) Ostwestry disability questionnaire (ODQ) (5) Modified Zung Questionnaire (6) Modified somatic perception questionnaire	Not reported	Consensus process not reported

*IMMPACT*: Initiative on Methods, Measurement, and Pain Assessment in Clinical Trials; ICF: International Classification of Functioning, Disability, and Health; VAPAIN: Validation and Application of a patient relevant core outcome set to assess effectiveness of multimodal pain therapy; # Recommended outcome domains for children and adolescents consist of Pain intensity, Global judgment of satisfaction with treatment, Symptoms and adverse events, Physical recovery, Emotional response, Economic factors.

**Table 2:** Recommendations for core outcome sets for daily record keeping in chronic pain and back pain.

Name of Initiative/Authors	(a) Condition (b) Intervention (c) Scope of application (d) Location	Core Outcome Set -Domains-	Core Outcome Set -Measurement Instruments-	Stakeholders	Additional Comments
**German Pain Questionnaire (DSF)** Casser et al. 2012 [42]	(a) Chronic pain (b) Specialized pain management, (c) Daily practice (d) Germany	several domains consisting of: (1) Patient’s demographic data of patient (2) Biographic data (3) Description of pain - Pain drawing and verbal description - Pain duration, frequency, course - Qualitative pain description - Pain intensity - Pain related disability - Causal- and control attribution (4) Psychological wellbeing - General wellbeing - Screening of anxiety, depression, and stress (5) Comorbidity (6) History of medical pretreatment - Physicians and interventions - medication	(1) self-report items (2) self-report items (3) self-report items, adaptation of the Brief Pain Inventory (BPI), Numerical Rating Scale for pain intensity (NRS,), Pain perception scale (SES), Chronic pain grade questionnaire (CPG) (4) Marburg Questionnaire of habitual wellbeing (MFHW), Depression-Anxiety-Stress-Scale (DASS) (5) self-report items (6) self-report items	Panel consisted of physicians specialized in pain medicine, psychotherapists, and researcher experienced in public health	Several updates Completed validation of the questionnaire Implementation in Germany via an electronical platform Benchmarking and observational studies by the German Pain Questionnaire supported Questionnaire supports diagnostic and therapeutic process
**Treatment Outcomes in Pain Survey (TOPS)** Rogers et al. 2000 [43] Rogers et al. 2000 [44]	(a) Chronic pain (b) Interdisciplinary pain management (c) Daily clinical care (d) International	**14 outcome domains** (1) Pain symptom (2) Perceived family/social disability (3) Objective family/social disability (4) Work limitations (5) Objective work disability (6) Lower body functional limitations (7) Upper body functional limitations (8) Fear avoidance (9) Passive coping (10) Life control (11) Solicitous responses (12) Patient satisfaction with care (13) Patient satisfaction with outcome (14) Total pain experience	A 120-item questionnaire constructed according to the identified domains for administration For follow-up were 61 items were provided	Not applicable	The tool was statistically derived from Short Form 36 (SF-36,), Multidimensional Pain Inventory (MPI), Oswestry Disability Questionnaire (ODQ and ), Brief pain Inventory (BPI) accomplished by several items to role-functioning, coping and pain (MOS)
**Treatment Outcomes in Pain Survey short version (S-TOPS)** Haroutiunian et al. 2012 [45]	(a) chronic pain (b) interdisciplinary pain management (c) daily clinical care/individual patient monitoring (d) international	7 outcome domains * (1) physical function lower body (2) physical function upper body (3) pain symptom (4) role-emotional disability (5) family and social disability (6) patient satisfaction with outcomes (7) patient satisfaction with care (for * please refer to *additional comments*)	Reanalyzes from original TOPS via factor analyzes Reducing the health related quality scale by replacing the original SF 36 by the shorter SF 12	Panel consisted of 11 clinicians (medical *n* = 4, physical therapy *n* = 3, behavioral medicine *n* = 2, pharmacotherapy *n* = 2) experienced in pain medicine Patients were asked about the acceptable length of the questionnaire	A multi-methodic approach has been conducted including judgement of experienced clinicians, defined criteria of psychometric property, inclusion of IMMPACT recommended domains (4/6), factor analyzes, and patients were asked about the acceptable amount of items for individual patient Aim of the tool is monitoring in multidisciplinary chronic pain treatment * To complete the recommended set of scales Haroutiunian et al. [34] suggested including two more scales performance/work disability scale and sleep scale
**Patient Centered Outcome Questionnaire (PCOQ)** Robinson et al. 2005 [48]	(a) Chronic pain (b) No specific (c) No specific (d) International	**4 outcome domains** Considering usual level, desired level and level of success for: (1) Pain (2) Fatigue (3) Emotional distress (4) Interference with daily activities	Single item assessment of (1) NRS pain (0–10) (2) NRS fatigue (0–10) (3) NRS emotional distress (0–10) (4) NRS interference with daily activities (0–10) For three levels: - usual level - desired level - level of success	Not reported	The identification of the domains and development of the scales was not clearly described Patient expectation were assessed for low back pain and fibromyalgia [50]
**Patient reported outcome criterion for operationalizing success in multi-modal pain therapy** Donath et al. 2015 [49]	(a) Chronic pain (b) Interdisciplinary multimodal pain therapy (c) Daily practice (d) Germany	**5 outcome domains** (1) Pain severity (2) Disability due to pain (3) Depressiveness (4) Physical health related quality of life (5) Mental health related quality of life	(1) Average pain severity (NRS 0–10) (2) Pain disability Index (PDI) (3) German version of the CESD (4) S-36,Short Form 26 (SF36), physical composite score (5) S-36,Short Form 26 (SF36), mental composite score	Not applicable	The tool was statistically derived from scales and items of the German Pain Questionnaire
